# Knowledge and perception of milk producers about thermal stress in Brazilian dairy farms

**DOI:** 10.1016/j.heliyon.2024.e26115

**Published:** 2024-02-17

**Authors:** Patrícia Kelly de Moraes Brettas, Fernanda Gatti de Oliveira Nascimento, Ednaldo Carvalho Guimarães, Priscila Neves Faria, Arthur Veiga Ferreira, Mara Regina Bueno de Mattos Nascimento

**Affiliations:** a Graduate Program in Veterinary Science, Faculty of Veterinary Medicine, Federal University of Uberlândia, Campus Glória, Bloco 1CCG, Sala 209 A e B, BR-050, KM 78, S/N, Uberlândia, Minas Gerais, CEP:38410-337, Brazil; b Faculty of Mathematics, Federal University of Uberlândia, Campus Santa Mônica, Bloco 1F, Sala 1F120, Av. João Naves de Avila, 2121, Bairro Santa Mônica, Uberlândia, Minas Gerais, CEP:38400-902, Brazil; c Presidente Antônio Carlos University, Unipac, Uberlândia, Minas Gerais, Brazil

**Keywords:** Dairy cows, Heat stress, Mitigation measures, Productivity, Questionnaire, Tropical environment

## Abstract

Heat stress is a challenge for the milk production chain, especially in tropical environments. Thus, the objective was to evaluate the knowledge and the perception of milk producers about heat stress and determine what corrective measures they adopted to minimize losses in the productive performance of dairy cows due to high temperatures. A questionnaire was applied to 112 dairy farmers in the states of Minas Gerais and Goiás, Brazil in 2019 and 2020. The collected data were submitted to descriptive statistics using the G test of independence and multivariate correspondence analysis. Among the surveyed producers, 89% stated that they had already been informed about what heat stress is and how it affects the animals; 94% declared that they had already noticed in their day-to-day activities that heat stress impairs productivity and milk quality, and 92% stated that they have tried to reduce the heat stress to which the animals in their herd are exposed. There was an association among previous knowledge about heat stress and farm size, daily volume of milk produced, number of lactating cows, type of milking and presence of technical assistance. There was also an association among the adoption of measures that minimize the negative effects of heat stress with the size of the property, the number of cows in lactation and with the producer's perception of heat stress in their daily lives. It was concluded that, in important municipalities of the Brazilian dairy chain, most milk producers surveyed have knowledge and perception of the negative effects of heat stress on the productive performance of dairy cows and sought to adopt measures that could alleviate them.

## Highlights

• Interviewed producers are knowledgeable about and have mitigated heat stress effects.
• With fewer lactating cows, producers perceive lower heat stress effects.
• Smaller properties have lower adoption of heat stress mitigation strategies.
• Without technical support and non-milk income, heat stress mitigation is limited.

## 1 Introduction

Changes and adverse global climate conditions generate great losses for global food production [1], and the prospects of climate changes warming are alarming with forecasts of prolonged periods of excessively hot weather, which will be an increasing challenge to maintain the health, well-being and productive efficiency of animals [2]. In the face of this threat, climate change and food security issues have been discussed by international organizations such as the World Health Organization and the Food and Agriculture Organization of the United Nations [3].

At the last international meeting to discuss climate change on the planet, which took place in November 2022, it was emphasized that geopolitical changes and extreme weather events can cause major disruptions in food supply chains, which harms the poorest in the world, intensifying hunger and malnutrition. Thus, it was advocated that food be produced in an inclusive, responsible and sustainable way [4].

When evaluating the negative impacts of climate changes specifically on the performance of dairy herds, heat stress is a major issue that represents a challenge for the sustainability of the milk production chain due to the increase in the frequency, duration and severity of events of intense heat resulting from climate change [5,6].

Heat stress is particularly associated with high air temperature and humidity and intense solar radiation, which compromises the animal's ability to maintain its homeothermy [7]. Consequently, thermal stress generates significant losses in the milk production chain, as it tends to compromise dry matter consumption, animal growth and well-being, milk production and quality, reproductive performance and immune responses of cows [6,8].

In addition, animals raised in pasture without shade, as is common for dairy cattle in Brazil, are more susceptible to heat stress due to intense solar radiation and high ambient temperature, and are therefore more vulnerable to climate change [9,10]. Thus, what was expected is that dairy farmers adopt measures to mitigate the effects of heat stress on dairy cows, such as greater availability of natural shading, which is the simplest and most economically viable way to provide thermal treatment and comfort for cattle raised on pasture [11].

The objective was to evaluate the knowledge and the perception of dairy farmers about heat stress and the harmful effects of high temperatures on the performance of dairy cows in an important milk producing region of Brazil (states of Minas Gerais and Goiás) and to identify the corrective measures that they effectively adopted in their production systems to minimize such losses.

## 2 Material and methods

This study was approved by the Research Ethics Committee (REC) of the Federal University of Uberlândia (UFU), under opinion number 3.506.229.

A questionnaire was applied to 112 dairy farmers from 17 municipalities in the states of Minas Gerais (MG) and Goiás (GO), Brazil, in 2019 and 2020. According to the Köppen classification, these regions have either the Cwa climate (hot subtropical climate with dry winter) or the Aw climate (tropical climate with dry winter) [12,13]. The consent was obtained from study participants before start to answer the survey. This questionnaire contained 11 questions and was classified as structured/semi-open, i.e., structured for presenting questions with pre-qualified answers (closed questions), and semi-open due to the closed answers that had the item “others”, which allowed explanatory observations when necessary [14].

The questionnaire was prepared according to the Knowledge, Attitude and Practice (KAP) research model, whose purpose was to establish a diagnosis on a given topic, helping to determine the effectiveness and/or the need for specific programs or interventions appropriate to the needs of the population [15]. In this context, knowledge refers to understanding, which is the ability to interpret a subject based on the acquisition and retention of information. Inversely, attitude was not directly related to an action, but rather to the positioning and organization of the individual's opinions in relation to a specific situation. Finally, practice was literally the actions of an individual in response to a stimulus, i.e., it is the decision-making to perform an action based on the application of rules and knowledge [15,16].

The questions were divided into categories that characterized the levels the participant's education and their production systems (size of the rural property, if milk is the main source of income, the volume of daily milk production, number of cows in lactation, dairy breeds used, type of milking used and if there is technical assistance). KAP assessments were focused on their Knowledge about heat stress (whether the producer has already been guided on what heat stress is and how it affects the animals), their Attitudes about the topic (e.g. “if you have noticed in your day-to-day life that heat stress impairs productivity and milk quality”) and their Practices, which in the present study referred to the adoption of mitigation measures (if the producer adopts measures to lessen the effects of heat stress to which the animals in your herd are exposed).

The application of the questionnaire was carried out based on direct face-to-face approaches, in dialogues involving questions and answers. Producers were selected by simple random sampling and the approach was individual. The sample size was calculated based on Equation (1) [17].

(1) $$n=\frac{\left(p\right)\;\left(q\right){\left(Z\right)}^{2}}{e^{2}}$$

where: n is the sample size (necessary number of producers to compose the sample), and p is the proportion with which the phenomenon occurs, i.e., the percentage of milk producers who have greater knowledge about heat stress and about the harm it can cause to the productivity of dairy cows and who adopt effective corrective measures in their systems production to minimize such losses. According to the literature [17], as the estimate of this proportion is unknown, “p” is replaced by 50% (0.5); q is the complementary proportion (1 – p); Z is the stipulated degree of confidence and e is the estimation of error.

It was considered 90% confidence (“Z” equal to 1.64) and an error of 8% (Equation (2)).

(2) $$n=\frac{\left(0.5\right)\;\left(0.5\right)\;{\left(1.64\right)}^{2}}{0.08^{2}}=105\;produtores$$

Therefore, the sample size obtained (112 dairy farmers) was greater than the minimum required.

The surveyed milk producers came from the municipalities of Araguari, Brejo Bonito, Campina Verde, Canápolis, Carmo do Paranaíba, Estrela do Sul, Frutal, Indianópolis, Lagoa Formosa, Monte Alegre, Paracatu, Patos de Minas, Prata, Presidente Olegário, Tupaciguara and Uberlândia, all from the mesoregion of Triângulo Mineiro/Alto Paranaíba, which is located in the western portion of the state of Minas Gerais and the Piracanjuba region in the state of Goiás, Brazil.

The data from the application of the questionnaire were submitted to descriptive statistics in Action®. The G test of independence (with 5% significance) was carried out in the BioEstat® 5.0 to elect the questionnaire results that showed a significant association with the knowledge and perception of milk producers about heat stress and with the adoption of measures of mitigation. Correspondence multivariate analysis was performed to generate groupings based on the association between two or more categorical variables (PAST®).

## 3 Results and discussion

With regard to education, 43% of respondents had completed primary education, followed by those who had completed secondary education (31%), higher education (20%) and, finally, technical education (6%). Borsanelli et al. [18], in a survey conducted with 171 dairy farmers from 96 municipalities in the State of São Paulo, found similar results. Conceicão et al. [19] reported a different education profile in a survey carried out in Corinto, a central region of Minas Gerais, where 56.7% had only completed elementary school but did not finish high, which demonstrates the variations that occur among the meso-regions in the state of Minas Gerais.

These results referring to schooling may represent a challenge in dairy activity, because, according to Oliveira et al. [20], producers with a lower level of education tend to have greater difficulty accepting new technologies and assimilating new information related to management practices.

The highest percentage of the size of dairy farms was between 50 and 100 ha (36%), followed by those between 10 and 50 ha (32%), over 100 ha (23%) and up to 10 ha (9%); therefore 77% of the properties had an area of up to 100 ha. Conceicão et al. [19] reported different stratification in a survey carried out in Corinto, Minas Gerais, where 70% of the properties had an area greater than 100 ha, which exemplifies the heterogeneity present in dairy farming in Brazil according to the region evaluated.

Among the surveyed producers, 69% had milk as their main source of income, a lower percentage than that found by Borsanelli et al. [18], in which 91.2% of the 171 producers surveyed (state and São Paulo) declared that dairy farming was their main activity. Therefore, the result found in the present research is worrying for the milk production chain in the region, because, according to Almeida and Silva [21], when the producer develops the dairy activity in a secondary way or in conjunction with others, its efficiency tends to be compromised by deficiencies in strategic planning; however, Wagner et al. [22], argued that the diversification of income sources actually allows small milk producers to maintain the activity.

Regarding productivity, 22% of the producers had a daily milk production of less than 50 L (L), 40% produced from 51 to 200 L/day and 38% had an average milk production greater than 200 L/day. These results were close to those found in a study with 171 dairy farmers in the State of São Paulo [18], most of whom (54.4%) had a daily production ranging from 50 to 200 L. Zeferino et al. [23], interviewing 92 producers in the north of MG (a semi-arid region), also reported a great heterogeneity of properties in relation to daily milk production, which ranged from 4.0 to 233.0 L/day/property.

Regarding the size of the herd, only 21% of the producers claimed to have more than 40 lactating cows, and therefore the majority of those surveyed had less than 40 lactating animals in their herds, with 4% of the properties having up to 5 lactating cows, 21% from 5 to 10, 7% from 10 to 25 and 47% from 25 to 40 lactating cows. These findings were similar to the research by Conceição et al. [19], in which 73.3% of the properties studied had less than 50 lactating cows. Gomes et al. [24], in a survey carried out in Vale do Jequitinhonha, MG, reported that 63.70% of the properties had up to 29 lactating cows.

According to the classification suggested by Zoccal et al. [10], there are four main types of dairy production systems: subsistence production, family-based production, semi-extensive production and specialized production. Thus, taking as a reference the size of the herd and the average daily milk production of most of the rural properties addressed, these would be classified as “production on a family basis”.

The highest percentage of racial composition was the Girolando and “crossbred” breeds, with different genetic compositions (Table 1). Next were the Holstein breed and Girolando on the same property. It is estimated that 80% of Brazilian milk production comes from crossbred dairy cows of the Girolando breed, originating from the crossing of the Holstein breed, which is of European origin and specialized in milk production with the Gyr (milkman) breed, which is of Indian origin [25]. The Girolando breed, which today has national and international recognition, was created seeking good productivity in tropical and subtropical regions, and there are different racial compositions, from 1/4 Holstein +3/4 Gyr to 7/8 Holstein +1/8 Gyr, However, a racial pattern is sought: 5/8 Holstein +3/8 Gyr [25].

**Table 1:** Breeds and genetic compositions of cows raised in 112 dairy farms, located in the states of Minas Gerais and Goias, Brazil, from 2019 to 2020.

Breeds	N (number of farms)	% (Percent)
Holstein	1	0.89
Holstein, Jersey and Girolando	1	0.89
Holstein, Jersey and UB^a^	1	0.89
Holstein, Gyr and Girolando	1	0.89
Holstein, Girolando and UB^a^	1	0.89
Brown Swiss	1	0.89
Gyr	1	0.89
Gyr and Girolando	1	0.89
Gyr, Girolando and Crossbred^b^	2	1.79
Girolando and UB^a^	2	1.79
Holstein, Crossbred^b^ and UB^a^	3	2.68
Jersey	3	2.68
UB^a^ (more than 2 breeds)	3	2.68
Jersey and Girolando	4	3.57
Jersey, Girolando and Crossbred^b^	4	3.57
Girolando	6	5.36
Crossbred^b^ (2 breeds)	14	12.50
Holstein and Girolando	22	19.64
Girolando and Crossbred^b^	41	36.61
Total	112	100

a UB (“undefined breed”): Animals without breed definition.

b Crossbred: progeny from the cross between an animal of European origin with another of indicine origin (Zebu group) (Embrapa Gado de Leite, 2003).

In addition, it is noted that few properties had herds consisting exclusively of one breed, such as Holstein, Brown Swiss, Gyr, Jersey, with a little more emphasis on herds composed only of cows of the Girolando breed.

Almost 80% of the 112 producers surveyed had Girolando animals in their herd, but a significant percentage of properties (58%) also raised crossbred cows. Usually, small milk producers opt for crossbred animals since they have greater resistance to challenges such as weather conditions and parasitic infestations, and in addition, the creation of European breeds specialized in milk production would require a high maintenance expense of the herd, such as nutritional and health management [20,26]

Regarding milking, 30% used mechanical milking with a bucket at the foot, 23% mechanical method in the milking parlour and almost half of the properties (47%) used manual milking, a result close to that of Conceição et al. [19] in the central region of MG, where 53.3% of the properties used manual milking. This result differed, however, from that found by Zeferino et al. [23], who recorded that 100% of the evaluated properties used the manual milking system, but it is important to emphasize that this last research was carried out in the semi-arid region of Minas Gerais (the northern regions of the state). According to Vieira et al. [27], manual milking is still widely used, especially because it has low costs, although the risk of milk contamination and milking time are higher. In addition, the choice of type of milking depends on the property's infrastructure, the number and productivity of animals and available labor.

Only 61% of the surveyed producers claimed to have technical assistance, a figure much lower than that found by Borsanelli et al. [18], who reported that about 92% of the surveyed producers (state and São Paulo) declared having veterinary assistance. Gomes et al. [24] also reported a higher percentage: 81.8% of producers received technical assistance.

Conversely, Conceição et al. [19] in a study in the central region of Minas Gerais, reported an even lower value than that found in the present study: Among those surveyed, only 23.3% received some type of technical assistance.

Technical guidelines on the determinants of milk production and farm management are important to improve production efficiency, especially for small milk producers [28]. In this context, it is important to emphasize that the milk production chain in Brazil has a large share of family-based production, whose low productivity and milk quality are associated with a lack of information, technical assistance and government and private investment [20].

Of all the producers surveyed, 100 (89%) stated that they had already been informed about what heat stress is and how it affects animals, especially dairy cows, which was categorized as knowledge on the subject. The other producers (12) denied having had access to this information. Pires et al. [29] evaluated the perception and responses to climate change (adoption of adaptive strategies) of rural producers in Minas Gerais and applied a questionnaire to 63 producers whose main sources of income were agriculture (40%) and livestock (38%). Similarly, the authors also found that the majority of those surveyed (89.65%) had already heard about and/or discussed climate change and its impact on agribusiness.

In addition, 105 (94%) of the 112 producers stated that they had already noticed in their daily lives that heat stress harms productivity and milk quality (attitude); six denied having this perception, and one did not know how to respond. This result was similar to that of Alves et al. [30], who, in a survey of 86 rural producers in the Zona da Mata region of Minas Gerais, recorded that 94% of those surveyed perceived changes in the climate throughout their lives and many of these producers pointed to temperature extremes, longer drought and less water availability as factors correlated with climate change that seemed to generate losses in animal production and were especially realized in weight loss, increased mortality and reduced milk production.

Similarly, Diniso et al. [3], in a survey of 106 workers on dairy farms in South Africa, mention that 92.5% of the participants stated that climate change harms the productivity of dairy cows, and 75.5% observed a reduction in the intake of matter dry when the air temperature is higher.

Finally, 103 (92%) producers stated that they try to reduce the heat stress to which the animals in their herd are exposed, and nine (8%) reported not adopting any measure. There were two producers who, despite having already received some guidance on heat stress and having already noticed losses in milk production due to heat stress, still did not adopt any measures that could alleviate it. Notably, all 12 producers who stated that they had never received information about what heat stress is and how it affects animals were still able to perceive in their routine with dairy cows the situations in which animals suffered negative effects from adverse thermal conditions because these were reflected in losses in milk production. All reported adopting measures to minimize the effects of heat stress on the herd.

Among the producers who did not adopt such mitigation measures, several justifications were presented, such as lack of knowledge about them, lack of financial resources for this purpose, lack of time and other specific reasons such as “Disincentive to run the farm due to age” or “The situation is difficult, making cheese takes a lot of time and there are no resources left to help with artificial shade for the cows”.

DiFalco et al. [31] cited the low frequency of technical assistance, lack of access to credit, or even information as possible factors that inhibit the adoption of adaptive measures. This statement was exemplified in the research by Pires et al. [29], in which, among producers with access to the internet (an important source of information), approximately 33% adopted measures to adapt to climate change, while among those who did not have internet, this value dropped to 14%.

Among the 103 producers who claimed to adopt measures to mitigate the effect of heat stress, it was found that the majority (74) simultaneously selected four or five options and only one selected a single option. This result can be considered positive because, according to Osei-Amponsah et al. [6], due to the intensity of climate change, the isolated adoption of one strategy is not enough; the ideal is a combination of mitigation strategies, such as providing shade associated with greater water availability, nutritional interventions and animal selection with a higher heat tolerance.

There was an association between previous knowledge about heat stress and the size of the property, the daily volume of milk produced, the number of cows in lactation, the type of milking and the presence of technical assistance (Table 2). There was no association between the milk producer's perception of heat stress in their routine and the previous questions (Table 3). Finally, there was an association between the adoption of measures that minimize the negative effects of heat stress with the size of the property, the number of lactating cows and the producer's perception of heat stress in their daily lives (Table 4).

**Table 2:** Associations between the questions that characterized the participants and their production systems with knowledge about heat stress, in a survey carried out with producers in Minas Gerais and Goiás, Brazil, from 2019 to 2020.

		Have you ever been told what heat stress is and how it affects animals, especially dairy cows?		
		No	Yes	Total
What is your level of school? (P-value = 0.334)	Elementary	8	40	48
	Middle	2	33	35
	Technical	1	6	7
	Superior	1	21	22
	Total	12	100	112
Property size in hectares? (P-value = 0.023)	up to 10 ha	3	7	10
	from 10 to 50 ha	7	30	37
	from 50 to 100	1	39	40
	over 100 ha	1	24	25
	Total	12	100	112
Is milk your main source of income? (P-value = 0.262)	Yes	6	71	77
	No	6	29	35
	Total	12	100	112
How many liters of milk are produced per day? (P-value<0.001)	Up to 50 L/dia	9	14	23
	Fron 51–200 L/dia	3	42	45
	Over 200 L/day	0	44	44
	Total	12	100	112
How many cows are lactating? (P-value = 0.003)	Up to 5 cows	2	2	4
	From 5 to 10 cows	7	17	24
	10 to 25 cows	1	7	8
	25 to 40 cows	2	51	53
	More than 40cows	0	23	23
	Total	12	100	112
What dairy breeds do you have in your herd? (P-value = 0.836)	Total	12	100	112
What type of milking method? (P-value = 0.002)	Manual	11	42	53
	Mechanical by foot	1	32	33
	Mechcanical in milking parlour	0	26	26
	Total	12	100	112
Do you have technical assistance from any agency or entity? (P-value = 0.031)	Yes	1	43	44
	No	11	57	68
	Total	12	100	112

P-value of independence G test (P-value <0.05, dependent attributes).

**Table 3:** Associations between the questions that characterized the participants and their production systems with the perception of heat stress, in a survey carried out with producers in Minas Gerais and Goiás, Brazil, from 2019 to 2020.

		Have you noticed in your day-to-day life that heat stress impairs milk productivity and quality?			
		No	Yes	I don't know	Total
What is your level of school? (P-value = 0.883)	Elementary	4	44	0	48
	Middle	1	33	1	35
	Technical	0	7	0	7
	Superior	1	21	0	22
	Total	6	105	1	112
Property size in hectares? (P-value = 0.071)	up to 10 ha	3	6	1	10
	from 10 to 50 ha	3	34	0	37
	from 50 to 100	0	40	0	40
	over 100 ha	0	25	0	25
	Total	6	105	1	112
Is milk your main source of income? (P-value = 0.755)	Yes	4	72	1	77
	No	2	33	0	35
	Total	6	105	1	112
How many liters of milk are produced per day? (P-value = 0.182)	Up to 50 L/dia	1	22	0	23
	Fron 51–200 L/dia	5	40	0	45
	Over 200 L/day	0	43	1	44
	Total	6	105	1	112
How many cows are lactating? (P-value = 0.639)	Up to 5 cows	1	3	0	4
	From 5 to 10 cows	3	21	0	24
	10 to 25 cows	1	6	1	8
	25 to 40 cows	1	52	0	53
	More than 40cows	0	23	0	23
	Total	6	105	1	112
What dairy breeds do you have in your herd? (P-value = 1)	Total	6	105	1	112
What type of milking method? (P-value = 0.288)	Manual	5	48	0	53
	Mechanical by foot	1	31	1	33
	Mechcanical in milking parlour	0	26	0	26
	Total	6	105	1	112
Do you have technical assistance from any agency or entity? (P-value = 0.375)	Yes	1	43	0	44
	No	5	62	1	68
	Total	6	105	1	112
Have you heard about what heat stress is and how it affects dairy cows? (P-value = 0.652)	Yes	6	93	1	100
	No	0	12	0	12
	Total	6	105	1	112

P-value of independence G test (P-value <0.05, dependent attributes).

**Table 4:** Associations between the questions that characterized the participants and their production systems with the adoption of strategies to mitigate their negative effects, in research carried out with producers in Minas Gerais and Goiás, Brazil, from 2019 to 2020.

		Do you try to reduce the heat stress your herd animals are exposed to?		
		No	Yes	Total
What is your level of school? (P-value = 0.788)	Elementary	4	44	48
	Middle	3	32	35
	Technical	0	7	7
	Superior	2	20	22
	Total	9	103	112
Property size in hectares? (P-value = 0.001)	up to 10 ha	4	6	10
	from 10 to 50 ha	5	32	37
	from 50 to 100	0	40	40
	over 100 ha	0	25	25
	Total	9	103	112
Is milk your main source of income? (P-value = 0.813)	Yes	6	71	77
	No	3	32	35
	Total	9	103	112
How many liters of milk are produced per day? (P-value = 0.598)	Up to 50 L/dia	1	22	23
	Fron 51–200 L/dia	5	40	45
	Over 200 L/day	3	41	44
	Total	9	103	112
How many cows are lactating? (P-value = 0.004)	Up to 5 cows	1	3	4
	From 5 to 10 cows	3	21	24
	10 to 25 cows	4	4	8
	25 to 40 cows	1	52	53
	More than 40cows	0	23	23
	Total	9	103	112
What dairy breeds do you have in your herd? (P-value = 0.754)	Total	9	103	112
What type of milking method? (P-value = 0.649)	Manual	5	48	53
	Mechanical by foot	3	30	33
	Mechcanical in milking parlour	1	25	26
	Total	9	103	112
Do you have technical assistance from any agency or entity? (P-value = 0.451)	Yes	2	42	44
	No	7	61	68
	Total	9	103	112
Have you heard about what heat stress is and how it affects dairy cows? (P-value = 0.572)	Yes	9	91	100
	No	0	12	12
	Total	9	103	112
Have you noticed in your day-to-day life that heat stress impairs milk productivity and quality? (P-value< 0.001)	Yes	2	103	105
	No	6	0	6
	I don't know	1	0	1
	Total	9	103	112

P-value of independence G test (P-value <0.05, dependent attributes).

When evaluating these associations, it is important to emphasize that among the 12 producers who stated that they had never received any guidance regarding heat stress, ten owned a property of up to 50 ha, nine reported an average daily production of up to 50 L of milk, nine had herds with up to 10 lactating cows, 11 producers used manual milking and 11 did not have access to technical assistance (Table 2).

Among the nine producers who stated that they did not adopt strategies that could minimize heat stress, all had a rural property of up to 50 ha, eight had herds with up to 25 lactating cows, and most (six producers) did not realize in their routine that the heat stress impairs productivity and milk quality or did not know how to reply to the questions (one producer) (Table 4).

All these correlated characteristics differed from most of the surveyed producers, as 59% of the evaluated properties had an area greater than 50 ha; 79% had an average milk production greater than 50 L/day; 68% of the herds had more than 25 cows in lactation; 53% adopted mechanical milking; 61% said they had technical assistance, and 94% said they had already noticed in their routine that heat stress impairs productivity and milk quality.

So, it can be inferred that the smaller the area of the property, the average daily production of milk and the number of lactating cows, the use of manual milking and the absence of technical assistance, so does the producer's knowledge about heat stress tend to be lower. Similarly, the smaller the property area, the number of lactating cows and the perception of the effects of heat stress on herd productivity, the smaller the adoption of strategies that minimize such negative effects tends to be.

The options most selected by the 103 dairy farmers who claimed to use techniques to reduce the negative effects of heat stress were providing more drinking water and providing more shade (natural and/or artificial) (Table 5). These two strategies are important to reduce losses in productivity and milk quality due to heat stress. Moreover, it is necessary to reflect on the size of the shaded area per animal, the quality of the shade, the distribution of shade, and water in the pasture.

**Table 5:** Options for measures that minimize the effects of heat stress in dairy herds presented and selected by the 103 producers who claimed to try to reduce the effects of heat stress to which the animals in their herd are exposed, in a survey carried out with producers in Minas Gerais and Goiás, Brazil, from 2019 to 2020.

Options	N^a^	%
a) Increased supply of drinking water;	100	97.1
b) Greater provision of shade (natural and/or artificial) for the cows;	96	93.2
c) Access of animals to lakes or ponds to cool down or provision of baths;	76	73.8
d) Changes in the nutritional management of the animals (time and type of food provided);	64	62.1
e) Preference for raising crossbred animals, more adapted to the heat;	59	57.3
f) Cooling of installations using fans, nebulizers or sprinklers;	18	17.5
g) Greater ceiling height to favor natural ventilation, adequate position of buildings (east-west) and painting of the roof;	25	24.3
h) Other (describe).	11	11.5

a N: Number of producers who selected the respective option.

During heat stress, dairy cows consume a lot more water, so they visit the drinker more frequently and spend time there [32]. This higher water consumption helps to promote body cooling and replace water losses resulting from the increase in respiratory rate and sweating that occur during thermal stress to promote greater heat dissipation into the environment through respiratory and cutaneous evaporative pathways, respectively [33,34].

In pastures, providing shade is also one of the most efficient and economically viable strategies to minimize the effects of heat stress [11]. Under grazing conditions, dairy cows exposed to heat stress seek shade, under which they tend to remain for a long time, especially when solar radiation increased [35]. Herbut et al. [36] emphasized that the shade generates a more pleasant microclimate for the animals due to the reduction of exposure to direct and diffuse shortwave solar radiation and for generating a decline in the ambient temperature. In addition, animals under shade may also have a reduction in radiant heat gain due to less long wave radiation emitted by shaded surfaces and, when lying down in shaded places and depending on the soil surface temperature, they may dissipate body heat into the ground by conduction [37].

However, the minimum dimensions recommended for the shaded area must be followed, preventing the animals from crowding together in the same shade, which would impair air circulation, thus reducing heat loss, especially by convection, in addition to generating competition for shaded area [37,38]. According to Silva and Maia [39], a cow must have at least 3 m^2^ of shade area.

The use of native trees helps sustainability by favoring carbon sequestration and mitigating the effects of heat stress, promoting greater thermal comfort for production animals, reducing their respiratory rate, body temperature and improving their performance. The research by Teixeira et al. [40], based on microclimatic data in an agroforestry system of four different trees native to the cerrado biome (Caryocar brasiliense, Solanum lycocarpum, Vochysia thyrsoidea and Pterodon emarginatus.), demonstrated the potential of their shade to reduce air temperature, the temperature of the soil and the Radiant Heat Load, in addition to the greater sequestration of carbon dioxide (CO2), since these tree species are able to absorb CO2 more efficiently from the atmosphere. The species that presented the best results was the tree *C. brasiliense* (local name: Pequi).

Option “h” (other measures) was selected by 11 producers (Table 5) and allowed them to describe other measures that are adopted and that were not described in the questionnaire. Some of the main responses were: “a well-wooded place”, “adoption of the Compost Barn system”, “extracting milk in the early morning and late afternoon”, “hose down the cows before milking as indicated by the veterinarian at the dairy".

**Fig. 1:**
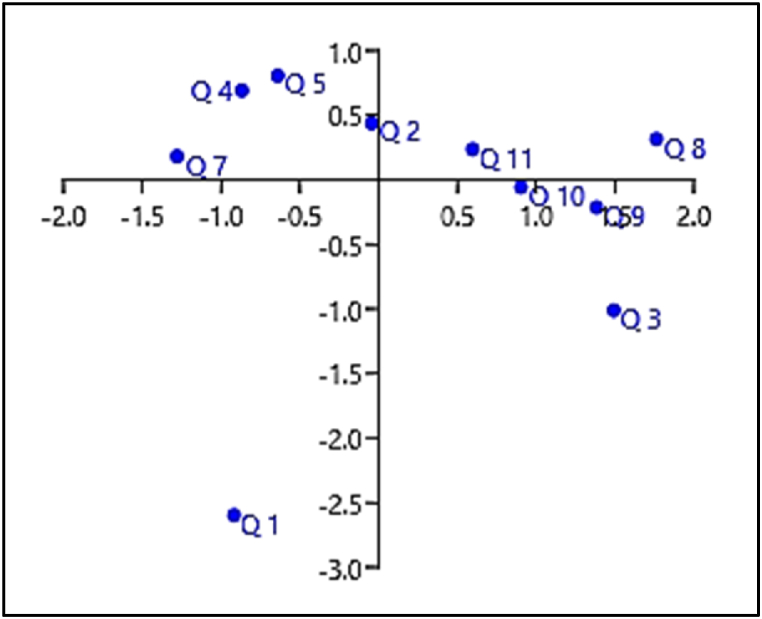
Bi-plot graph generated by the multivariate analysis of correspondence of the 11 questions of the questionnaire applied to 112 milk producers in Minas Gerais and Goiás, Brazil, from 2019 to 2020. Q1 (Question 1) - the education level; Q2 (Question 2) - size of the rural property; 3 (Question 3) - if milk is the main source of income; Q4 (Question 4) - the volume of daily milk production; Q5 (Question 5) - number of cows in lactation; Q6 (Question 6) - dairy breeds used; Q7 (Question 7) - type of milking used; Q8 (Question 8) - if there is technical assistance; Q9 (Question 9) - whether the producer has already been guided on what heat stress is and how it affects the animals; Q10 (Question 10) - their attitudes about the topic (e.g. “if you have noticed in your day-to-day life that heat stress impairs productivity and milk quality”); Q11 (Question 11) - if the producer adopts measures to lessen the effects of heat stress to which the animals in your herd are exposed.

Finally, Fig. 1 and Table 6 show the multivariate correspondence analysis, with principal components 1, 2 and 3 able to explain about 76% of the variability contained in the data set. Principal component 1 explained 38% of the data set and the questions that most contributed to its definition were questions 8, 3, 9, 10 and 11, which reinforces the correct structuring of the questionnaire, whereas questions 9, 10 and 11 were those that most contributed to the achievement of the research objective. In addition, the multivariate analysis of correspondence also indicated an association between the presence of technical assistance (question 8), having milk as the main source of income (question 3), guidance on heat stress (question 9), perception in routine on the impact of thermal stress (question 10) and the adoption of measures to mitigate its damage (question 11).

**Table 6:** Autovalues generated by the multivariate correspondence analysis.

Principal Components	Autovalue	% of total	Cumulative
1	0.0392071	38.086	38.096
2	0.024902	24.196	62.292
3	0.0142635	13.859	76.151
4	0.00750703	7.2943	83.446
5	0.00605421	5.8826	89.328
6	0.0038562	3.7469	93.075
7	0.00333483	3.2403	96.315
8	0,.00227,167	2.2073	98.523
9	0.00152046	1.4774	100

## 4 Conclusion

In important municipalities of the Brazilian dairy chain, most milk producers surveyed have knowledge and perception of the negative effects of heat stress on the productive performance of dairy cows, and therefore seek to adopt measures that can alleviate them, such as a greater supply of water and shade for the animals.

## Declarations

This study was approved by the Research Ethics Committee (REC) of the Federal University of Uberlândia (UFU), under opinion number 3.506.229.

## Funding

This study was partly financed by the 10.13039/501100002322Improvement Coordination of Higher Education Personnel – Brazil (CAPES).

## Data availability

Data will be made available on request.

## CRediT authorship contribution statement

**Patrícia Kelly de Moraes Brettas:** Writing – review & editing, Writing – original draft, Visualization, Validation, Resources, Project administration, Methodology, Investigation, Formal analysis, Data curation, Conceptualization. **Fernanda Gatti de Oliveira Nascimento:** Writing – original draft, Visualization, Validation, Supervision, Resources. **Ednaldo Carvalho Guimarães:** Writing – original draft, Visualization, Validation, Supervision, Resources, Investigation, Data curation. **Priscila Neves Faria:** Writing – original draft, Visualization, Validation, Supervision, Methodology, Data curation. **Arthur Veiga Ferreira:** Writing – original draft, Data curation. **Mara Regina Bueno de Mattos Nascimento:** Writing – original draft, Visualization, Validation, Supervision, Project administration, Methodology, Investigation, Formal analysis, Data curation, Conceptualization.

## Declaration of competing interest

The authors declare that they have no known competing financial interests or personal relationships that could have appeared to influence the work reported in this paper.
