# Supplementary material for: Knowledge and perception of milk producers about thermal stress in Brazilian dairy farms
Source: Heliyon. 2024 Feb 17;10(4):e26115. doi: 10.1016/j.heliyon.2024.e26115 (PMC10884848; doi:10.1016/j.heliyon.2024.e26115)
Supplement: Multimedia component 1 [file mmc1.docx]

| **QUESTIONNAIRE**  1. What is your education level?  1. ( ) Elementary  2. ( ) Medium  3. ( ) Technician  4. ( ) Superior  2. Property size in hectares?  1. ( ) up to 10 hectares  2. ( ) from 10 to 50 hectares  3. ( ) from 50 to 100 hectares  4. ( ) over 100 hectares  3. Is milk your main source of income?  1. ( ) Yes  2. ( ) No  4. How many liters of milk are produced per day?  1. ( ) up to 50 liters/day  2. ( ) from 51 to 200 liters/day  3. ( ) over 200 liters/day (Amount: _____ liters/day)  5. How many cows are lactating?  1. ( ) up to 5 cows  2. ( ) from 5 to 10 cows  3. ( ) from 10 to 25 cows  4. ( ) from 25 to 40 cows  5. ( ) more than 40 cows  6. What dairy breeds do you have in your herd?  1. ( ) Holstein  2. ( ) Swiss Brown  3. ( ) Jersey  4. ( ) Gyr  5. ( ) Girolando  6. ( ) Crossbreed animals  7. ( ) UB (“undefined breed”): Animals without breed definition  7. What type of milking?  1. ( ) Manual  2. ( ) Foot mechanics  3. ( ) Mechanics in the milking parlor  8. Do you have technical assistance from any agency or entity?  1. ( ) Yes  2. ( ) No  9. Have you ever been told what heat stress is and how it affects animals, especially dairy cows?  1. ( ) Yes  2. ( ) No  10. Have you noticed in your day-to-day life that heat stress impairs milk productivity and quality?  1. ( ) Yes  2. ( ) No  3. ( ) I don't know  11. Do you try to reduce the heat stress your herd animals are exposed to?  1. ( ) **NO.**  **Why?**  a) ( ) Lack of knowledge about the subject;  b) ( ) Lack of financial resources;  c) ( ) Other reasons. Which?____________________________________________________________  2. ( ) **YES.**  **How?**  a) ( ) Increased supply of drinking water;  b) ( ) Greater provision of shade (natural and/or artificial) for the cows;  c) ( ) Access of animals to lakes or ponds to cool down or provision of baths;  d) ( ) Changes in the nutritional management of the animals (time and type of food provided);  e) ( ) Preference for raising crossbred animals more adapted to the heat;  f) ( ) Cooling of facilities using fans, nebulizers or sprinklers.  g) ( ) Changes in the infrastructure of the facilities: higher ceiling height to favor natural ventilation, more suitable position of the buildings (east-west) and painting of the roof;  h) ( ) Other measures. Which?__________________________________________________________ |
| --- |
